# Therapeutic effect of osimertinib plus cranial radiotherapy compared to osimertinib alone in NSCLC patients with EGFR-activating mutations and brain metastases: a retrospective study

**DOI:** 10.1186/s13014-021-01955-7

**Published:** 2021-12-05

**Authors:** Xiaoyang Zhai, Wanhu Li, Ji Li, Wenxiao Jia, Wang Jing, Yaru Tian, Shuhui Xu, Yuying Li, Hui Zhu, Jinming Yu

**Affiliations:** 1 Shantou University Medical College, Shantou, 515041 Guangdong Province China; 2 Department of Radiation Oncology, Shandong Cancer Hospital and Institute, Shandong First Medical University and Shandong Academy of Medical Sciences, 440 Jiyan Road, Jinan, 250117 Shandong Province China; 3 Department of Radiology, Shandong Cancer Hospital and Institute, Shandong First Medical University and Shandong Academy of Medical Sciences, Jinan, 250117 Shandong Province China; 4 Department of Oncology, Renmin Hospital of Wuhan University, Wuhan, 430060 Hubei China; 5 Department of Radiation Oncology, Shandong Cancer Hospital and Institute Affiliated to Shandong University, Shandong First Medical University, Shandong Academy of Medical Sciences, Shandong Province 250117 Jinan, China

**Keywords:** Osimertinib, Cranial radiotherapy, Brain metastases, Leukoencephalopathy, Non-small cell lung cancer

## Abstract

**Background:**

The study aimed to compare the efficacy of osimertinib plus cranial radiotherapy (RT) with osimertinib alone in advanced non-small-cell lung cancer (NSCLC) patients harboring epidermal growth factor receptor (EGFR) mutations and brain metastases (BMs).

**Methods:**

The clinical data of advanced NSCLC patients with BMs who received osimertinib were retrospectively collected. The patients were assigned to one of the two groups according to the therapeutic modality used: the osimertinib monotherapy group or the osimertinib plus RT group.

**Results:**

This was a retrospective study and 61 patients were included from December 2015 to August 2020. Forty patients received osimertinib monotherapy, and twenty-one patients received osimertinib plus RT. Radiotherapy included whole-brain radiation therapy (WBRT, *n* = 14), WBRT with simultaneous integrated boost (WBRT-SIB, *n* = 5) and stereotactic radiosurgery (SRS, *n* = 2). The median number of prior systemic therapies in the two groups was one. Intracranial and systemic ORR and DCR were not significantly different between the two groups. No difference in iPFS was observed between the two groups (median iPFS: 16.67 vs. 13.50 months, *P* = 0.836). The median OS was 29.20 months in the osimertinib plus RT group compared with 26.13 months in the osimertinib group (HR = 0.895, *P* = 0.826). In the L858R mutational subgroup of 31 patients, the osimertinib plus RT group had a longer OS (*P* = 0.046). In the exon 19 deletion mutational subgroup of 30 patients, OS in the osimertinib alone group was longer than that in the osimertinib plus RT group (*P* = 0.011). The incidence of any-grade adverse events was not significantly different between the osimertinib plus RT group and the osimertinib alone group (47.6% vs. 32.5%, *P* = 0.762). However, six patients (28.5%) experienced leukoencephalopathy in the osimertinib plus RT group, and 50% (3/6) of the leukoencephalopathy was greater than or equal to grade 3.

**Conclusion:**

The therapeutic effect of osimertinib with RT was similar to that of osimertinib alone in EGFR-positive NSCLC patients with BM. However, for patients with the L858R mutation, osimertinib plus RT could provide more benefit than osimertinib alone.

## Background

The incidence of brain metastases (BMs) in NSCLC patients was reported to be 25% to 30% during the treatment process [1, 2]. For patients harboring epidermal growth factor receptor (EGFR) mutations, the rate can even reach 39.2% [3].

In the era of chemotherapy, agents with large molecular weights have difficulty crossing the blood–brain barrier (BBB), causing poor prognosis in patients with brain metastases [4]. With the development of targeted therapy, EGFR tyrosine kinase inhibitors (TKIs) with small molecular weights have replaced chemotherapy as first-line treatment for advanced NSCLC patients with EGFR mutations. The permeation ratios of the BBB in gefitinib and erlotinib were approximately 1% and 4.5%, respectively [5, 6]. Although the permeation ratio of the BBB remains limited, the efficacy for patients with BM has improved. A phase II trial indicated that in lung adenocarcinoma patients with BM and EGFR mutations receiving gefitinib, the median intracranial progression-free survival (iPFS) was 14.5 months [7]. The CTONG-0803 study enrolling patients with advanced NSCLC and asymptomatic BMs suggested that erlotinib as second-line therapy has an iPFS of 10.1 months [8]. Moreover, LUX-Lung 6 showed that in patients with baseline BMs and EGFR mutations, the median time to central nervous system (CNS) progression in the afatinib group was 7.9 months longer than that in the chemotherapy group [9].

Cranial radiotherapy (RT) includes whole-brain radiation therapy (WBRT), stereotactic radiosurgery (SRS) and WBRT with simultaneous integrated boost (WBRT-SIB). Nowadays, SRS has become standard of care for patients with BMs. Not only for patients with oligo-BMs, but also for patients with 4–10 BMs with a cumulative tumor volume of fewer than 15 ml, SRS could also be recommended. WBRT, as an alternative scheme, could be selected for patients with multiple BMs not eligible for SRS, in line with performance status, number, and location of BMs, and neural symptoms [10]. Nevertheless, WBRT is the primary radiotherapy method for patients with leptomeningeal metastases (LM), though whether it could extend survival remains debatable.

Previous studies indicated that RT has a synergistic effect with TKIs. Cranial radiotherapy can promote TKI crossing of the BBB and improve the drug concentration of TKIs in cerebrospinal fluid (CSF), while TKIs can enhance the antitumor effect of radiotherapy by radiation sensitization [11]. A meta-analysis involving 363 NSCLC patients with EGFR mutations and BMs to compare the efficacy between upfront cranial radiotherapy and TKI alone found that upfront radiotherapy improved four-month iPFS and two-year overall survival (OS) [12]. However, cranial radiotherapy, especially WBRT, may cause CNS toxicity, such as hypomnesia or leukoaraiosis. With prolonged survival of patients with BMs, the adverse events of RT have attracted increasing attention.

Osimertinib, as a third-generation EGFR-TKI, has a higher permeation ratio of the BBB than other TKIs in a preclinical study [13]. The FLAURA study indicated that osimertinib as a first-line therapy extended the median progression-free survival (PFS) by 5.6 months compared with gefitinib or erlotinib in NSCLC patients with CNS metastases [14]. Although some studies reported that gefitinib or erlotinib plus RT was better than gefitinib or erlotinib alone [12, 15], whether osimertinib combined with RT is superior to osimertinib alone remains unknown. Therefore, we performed a retrospective study to explore the therapeutic effect and safety of osimertinib plus RT and further compared the clinical outcomes of osimertinib plus RT with those of osimertinib alone in NSCLC patients with EGFR-activating mutations and BMs.

## Patients and methods

### Study design and patients

We retrospectively reviewed the medical records of advanced NSCLC patients who were initially diagnosed with BM or progressed from BM and received osimertinib in Shandong Cancer Hospital and Institute (Jinan, Shandong, China) between December 2015 and August 2020. The inclusion criteria were as follows: (1) histologically or cytologically confirmed lung adenocarcinoma; (2) BM identified by magnetic resonance imaging (MRI), including cytologically or radiographically diagnosed LM; (3) patients harboring exon 19 deletion or the L858R mutation; (4) patients receiving concurrent RT or sequential RT with osimertinib. Patients harboring a negative T790M mutation or previously receiving osimertinib and RT were excluded. The study was approved by the Ethics Committee of Shandong Cancer Hospital and Institute. All procedures involving patients conformed to the principles outlined in the Declaration of Helsinki.

### Treatment protocol

According to whether brain radiotherapy was added, the patients were divided into the osimertinib group and osimertinib plus RT group. The dose of osimertinib was 80 mg once a day. Radiotherapy in the osimertinib plus RT group included WBRT (30 Gy in 10 fractions and 3 Gy per fraction), WBRT-SIB (30 Gy in 10 fractions and 3 Gy per fraction for whole brain with additional 10–20 Gy for BM) and SRS (30–45 Gy in 5–10 fractions). WBRT was used for patients with multiple BMs (> 3), LM and not eligible for SRS. For single or concentrated lesions, WBRT-SIB was worthy of consideration for improving local control rate. Due to the lack of sophisticated equipment, such as magnetic resonance simulator (MR-Sim), SRS was mainly applied to patients with 1–3 BMs in our study.

Patients receiving concurrent osimertinib and RT or sequential osimertinib and RT were enrolled. Concurrent radiotherapy referred to receiving osimertinib at the beginning of cranial radiotherapy, and sequential radiotherapy referred to receiving osimertinib after the end of RT. The interval between the end of RT and the beginning of osimertinib was less than a week.

The final treatment regimen was decided by the attending physician under their clinical experience and patient characteristics, including patient age, general condition, burden of BM, location of BM and personal willingness. Generally, for patients with asymptomatic BM or LM, advanced age, no intention to undergo RT and intolerance to radiotherapy, osimertinib alone was selected. For patients with symptomatic BM and LM, a high burden of brain metastases and tolerance for RT, cranial radiotherapy was recommended.

### Assessment of response and toxicity

Systemic and intracranial tumor responses were mainly evaluated by contrast-enhanced computed tomography (CT) scans and contrast-enhanced brain MRI scans. In the osimertinib alone group, tumor response was routinely evaluated once every 2–3 months from the beginning of osimertinib. In the osimertinib plus RT group, systemic and intracranial response evaluations were performed 1 month after completion of RT and then once every 2–3 months thereafter. Lumbar puncture and CSF cytology were recommended for the evaluation of LM, but they were not compulsive. If intracranial or systemic progression was suspected, contrast-enhanced brain MRI or CT was performed in time. The systematic treatment response was assessed in accordance with the Response Evaluation Criteria in Solid Tumors (RECIST) version 1.1. The intracranial response was evaluated by the Neuro-Oncology Brain Metastases Criteria (RANO-BM) and Neuro-Oncology leptomeningeal Metastases Criteria (RANO-LM). Adverse events (AEs) were evaluated and graded based on the National Cancer Institute Common Terminology Criteria for Adverse Events (CTCAE) version 4.0. Leukoencephalopathy, as an adverse event of WBRT, was estimated by Fazekas grade and sore.

### Endpoints

The primary endpoint was iPFS. Intracranial PFS referred to the time from the treatment initiation of osimertinib to intracranial progression or death. No intracranial progression was regarded as censored data at the last follow-up. The secondary endpoints included the intracranial objective response rate (ORR), intracranial disease control rate (DCR), PFS and OS. Intracranial ORR refers to the proportion of patients who achieve complete response (CR) and partial response (PR) of intracranial lesions. Intracranial DCR referred to the proportion of patients who had CR, PR and stable disease (SD) of intracranial lesions. PFS was measured as the time interval from the initiation of osimertinib to systemic progression, death from any cause or the last known follow-up. OS was defined as the time interval from the initiation of osimertinib to death caused by any reason or the last known follow-up.

### Statistical analysis

All statistical analyses were performed by using GraphPad Prism software version 8.0 (GraphPad Software, Inc., USA) and SPSS statistical software version 20.0 (IBM Corp., USA). The comparisons of patients’ baseline characteristics, tumor response rate and AEs in the two groups were analyzed by using the Chi-square test and Fisher’s exact test. The Kaplan–Meier method was used to calculate iPFS, PFS and OS. The difference in survival curves between the two groups was estimated by the log-rank test. Two-sided *P* values < 0.05 were considered statistically significant.

## Results

### Patient characteristics

Between December 2015 and August 2020, 121 NSCLC patients with BM and EGFR 19 del or L858R mutations received osimertinib in our cancer center. Thirty-four patients were excluded for using osimertinib initially because of extracranial progression, 20 patients were excluded for previous cranial radiotherapy, and 6 patients were excluded for T790M-negative mutation. As a result, 61 patients who received osimertinib were enrolled in the study. All enrolled patients had stage IV lung adenocarcinoma. According to the therapeutic modality, there were 40 patients in the osimertinib group and 21 patients in the osimertinib plus RT group. The last follow-up date was August 16, 2020. Thirty patients were still alive, and seventeen patients had died by the end of the follow-up. Fourteen patients were lost to follow-up, and the follow-up rate was 77.0%. The median follow-up time was 15.3 months (range, 3.0–43.0 months) for all patients.

The baseline characteristics of all patients in the two groups are presented in Table 1. There were no differences in the distribution of all variables between the two groups. The median age of patients in the two groups was 54 years, and the age range was 33 to 74 years. Thirty-one (50.8%) were male, while thirty (49.2%) were female. Twenty-seven (44.3%) patients had developed leptomeningeal metastases, and forty-eight (78.6%) patients were never smokers. Fifty-six (91.8%) patients had extracranial metastases, and 38 (62.2%) patients had more than three lesions of brain metastases. The rate of symptomatic patients was 57.1% in the osimertinib plus RT group and 52.5% in the osimertinib group.

**Table 1:** Patient characteristics

Characteristic	Total	Osimertinib group		Osimertinib + RT group		*χ*2	*p*
		No	%	No	%		
Gender							
Male	31	23	57.5	8	38.1		
Female	30	17	42.5	13	61.9	2.075	0.150
Age (years)							
Range	33–74	37–74		33–65			
Median	54	51.5		55			
< 65	52	32	80.0	20	95.2		
≥ 65	9	8	20.0	1	4.8	2.542	0.146
Smoking status							
Smoker	13	7	17.5	6	28.6		
Never smoked	48	33	82.5	15	71.4	1.007	0.341
Extracranial lesions							
Yes	56	37	92.5	19	90.5		
No	5	3	7.5	2	9.5	0.075	1.000
Brain metastases							
Symptomatic	33	21	52.5	12	57.1		
Asymptomatic	28	19	47.5	9	42.9	0.120	0.730
Number of brain metastases							
≤ 3	23	15	37.5	8	38.1		
**> 3**	38	25	62.5	13	61.9	0.002	0.964
Leptomeningeal metastases							
Yes	27	20	50.0	7	33.3		
No	34	20	50.0	14	66.7	1.550	0.213
EGFR mutation							
Exon 19 deletion	30	20	50.0	10	47.6		
21 L858R	31	20	50.0	11	52.4	0.031	0.860
T790M mutation							
Positive	51	33	82.5	18	85.7		
Unknown	10	7	17.5	3	14.3	0.104	1.000
Range	0–5	0–4		0–5			
Median	1	1		1			
0	7	4	10.0	3	14.3		
1	34	23	57.5	11	52.4		
> 1	20	13	32.5	7	33.3	0.449	0.927
Prior lines of TKI therapy							
Range	0–2	0–2		0–1			
Median	1	1		1			
0	8	4	10.0	4	19.0		
1	52	35	87.5	17	81.0		
2	1	1	2.5	0	0	1.521	0.628

*RT* cranial radiotherapy, *χ*2 Chi-square statistic, *EGFR* epidermal growth factor receptor, *TKI* tyrosine kinase inhibitors

Thirty (49.2%) patients had EGFR 19 del, and 31 (50.8%) patients had the EGFR L858R mutation. Fifty-one had T790M-positive mutations, and ten had an unknown T790M type. Four of these ten patients received osimertinib as the first-line therapy and the other six patients received osimertinib as second- or third-line treatment. For these six patients, rejection of biopsy due to economic factors or patients' inability to tolerate rebiopsy was the main reason for unknown T790M.

The number of prior systemic therapies between the two groups was not significantly different. The median number of prior systemic therapies in the two groups was one. The ranges were 0 to 4 in the osimertinib group and 0 to 5 in the osimertinib plus RT group. In all patients, 34 (55.7%) patients received osimertinib as second-line therapy, and 20 (32.8%) patients used osimertinib as third-line or later treatment. 86.6% of patients previously received first- or second-generation EGFR TKIs, of which 77.5% were erlotinib or gefitinib.

In 21 patients treated with osimertinib plus RT, 17 (80.9%) patients received osimertinib and radiation concurrently, and 4 (29.1%) patients received sequential treatment. Cranial radiotherapy included WBRT, WBRT-SIB and SRS. Fourteen patients received WBRT, 5 patients received WBRT-SIB, and 2 patients received SRS.

### Response evaluation

According to RANO criteria, in all patients, the intracranial ORR was 40.9%, and the intracranial DCR was 96.7%. The intracranial response rates were 38.1% and 42.5% in the osimertinib plus RT group and osimertinib group, respectively (*P* = 0.740). Among patients who received osimertinib plus RT, 2 patients (9.5%) achieved complete response of the intracranial metastases. The DCR of intracranial tumors was 95.0% and 100% in the two groups, respectively (*P* = 0.541). The intracranial tumors of all patients in the osimertinib plus RT group achieved effective control. However, intracranial ORR and DCR were not significantly different between the two groups. Based on RECIST v1.1, the systemic ORR was 21.3%, and the DCR was 91.8%. The systemic ORR and DCR were not significantly different between the two groups. The systemic ORR was 28.5% in the osimertinib plus RT group and 17.5% in the osimertinib alone group (*P* = 0.341). The systemic DCR was 90.5% and 92.5% in the osimertinib plus RT group and osimertinib alone group, respectively (*P* = 1.000) (Table 2).

**Table 2:** Intracranial response and systemic response in patients with NSCLC and brain metastases in the osimertinib group or osimertinib plus RT group

	Intracranial response (RANO criteria)		Systemic response (RECIST v1.1)	
	Osimertinib alone (*n* = 40)	Osimertinib + RT (*n* = 21)	Osimertinib alone (*n* = 40)	Osimertinib + RT (*n* = 21)
Objective response, *n* (%)	17(42.5%)	8(38.1%)	7(17.5%)	6(28.5%)
* P* value	0.740		0.341	
Disease control rate, *n* (%)	38(95.0%)	21(100.0%)	37(92.5%)	19(90.5%)
* P* value	0.541		1.000	
Best overall response, *n* (%)				
Complete response	3(7.5%)	2(9.5%)	0	0
Partial response	14(35.0%)	6(28.6%)	7(17.5%)	6(28.6%)
Stable disease	21(52.5%)	13(61.9%)	30(75.0%)	13(61.9%)
Progressive disease	2(5.0%)	0	3(7.5%)	2(9.5%)

*RT* cranial radiotherapy

### Survival evaluation

**Fig. 1:**
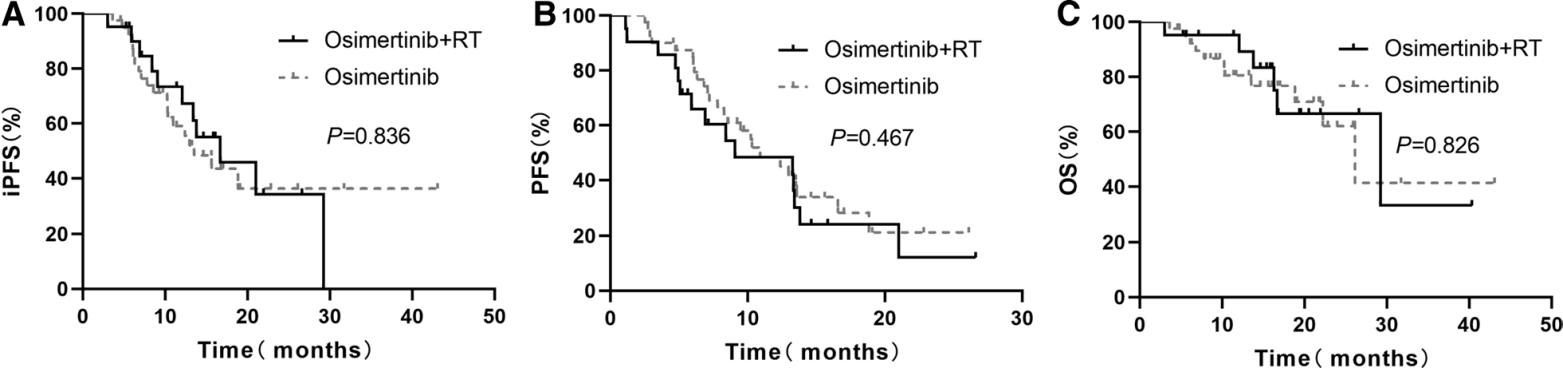
Survival outcomes of the patients in the two groups. **a** Intracranial PFS. **b** Systemic PFS. **c** OS

The median iPFS was 16.67 months in the osimertinib plus RT group and 13.50 months in the osimertinib alone group. No significant differences in iPFS were observed between the two groups (*P* = 0.836, Fig. 1a). The median PFS was 9.0 months and 10.9 months in the osimertinib plus RT group and osimertinib group, respectively (*P* = 0.467, Fig. 1b). The PFS rates at 1 year in the combination therapy group and monotherapy group were 42.3% and 45.0%, respectively. The median OS times in the osimertinib plus RT group and osimertinib group were 29.20 months and 26.13 months, respectively. The OS rates at 2 years in the combination therapy group and monotherapy group were 66.6% and 62.0%, respectively. OS was not significantly different between the two groups (*P* = 0.826, Fig. 1c).

**Fig. 2:**
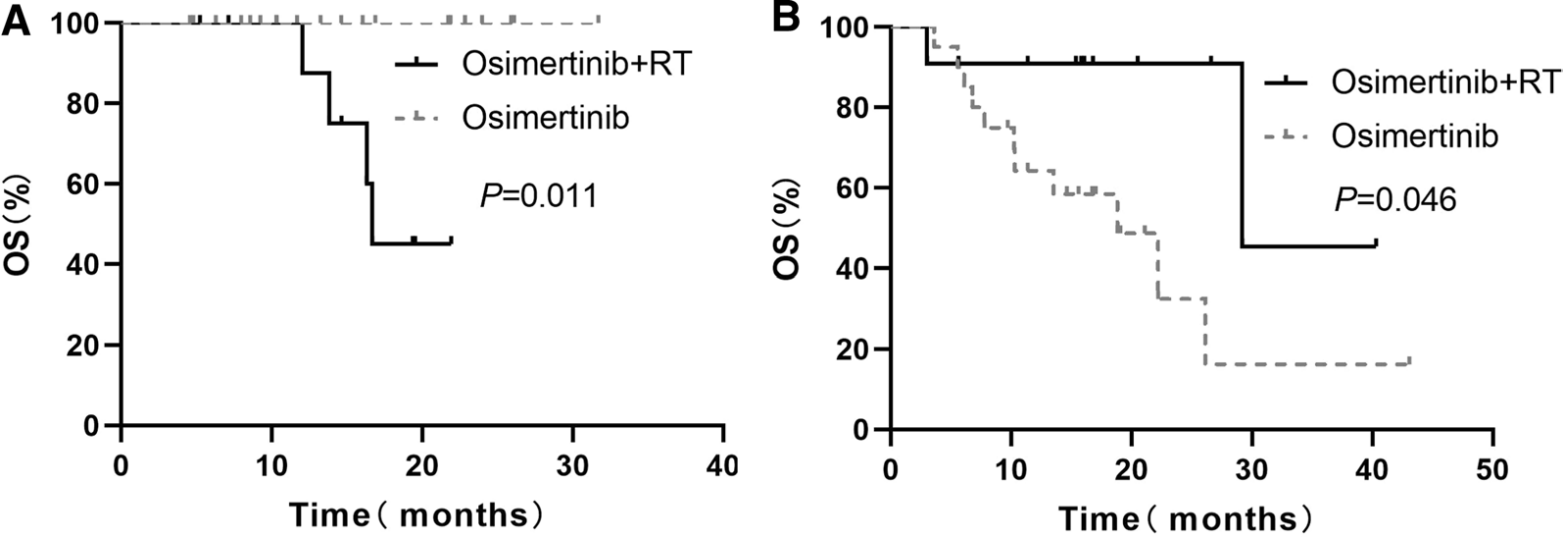
OS of the patients with EGFR 19del or L858R between the two groups. **a** EGFR 19del. **b** L858R

**Fig. 3:**
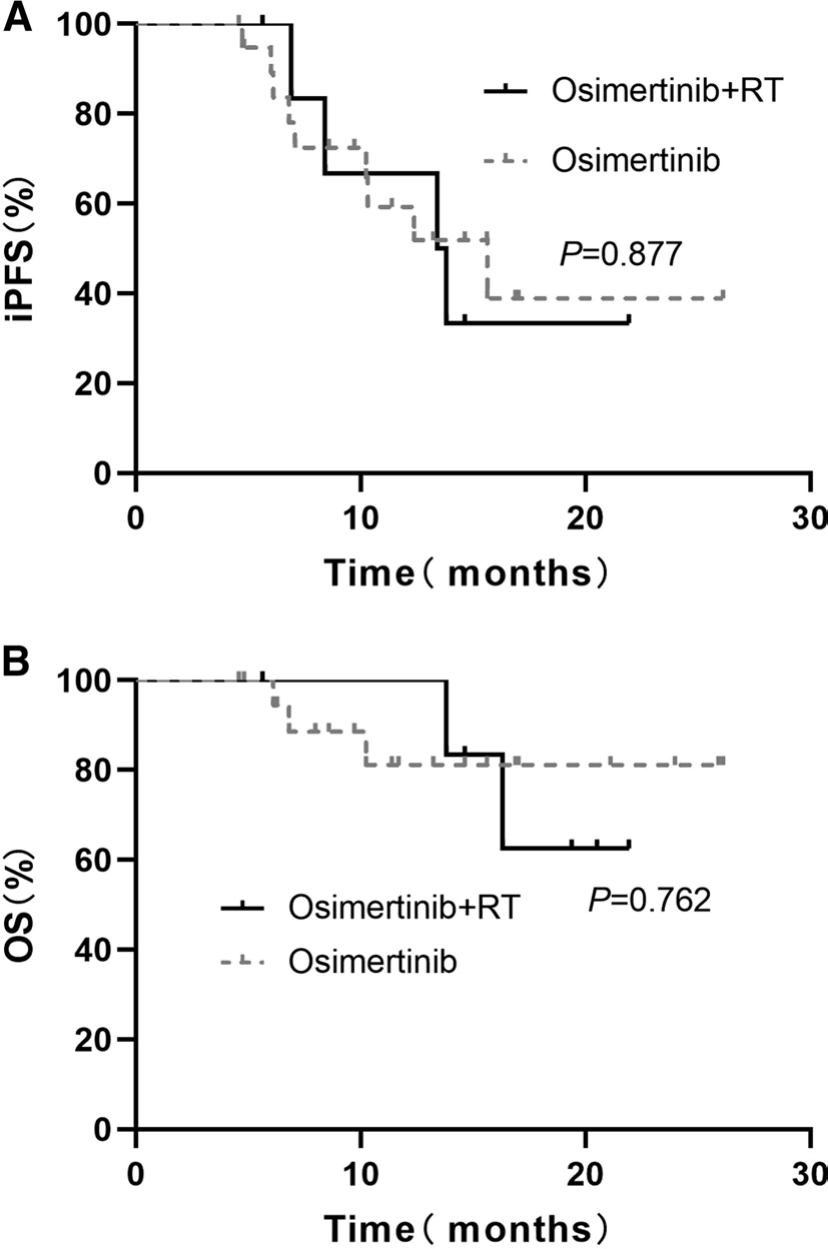
Survival outcomes of the patients with leptomeningeal metastases between two groups. **a** Intracranial PFS. **b** OS

In the subgroup of 30 patients with EGFR 19del, the OS was significantly longer in the osimertinib group than in the osimertinib plus RT group. The median OS was 16.6 months in the osimertinib plus RT group and was not reached in the osimertinib group (*P* = 0.011, Fig. 2a). In the subgroup of 31 patients with L858R, the OS was significantly longer in the osimertinib plus RT group than in the osimertinib group. The median OS was 29.2 months in the osimertinib plus RT group and 18.8 months in the osimertinib group (*P* = 0.046, Fig. 2b). In the subgroup analysis for 27 patients with leptomeningeal metastases, the iPFS and OS between the osimertinib plus RT and osimertinib alone groups were not significantly different (iPFS, 13.60 months vs. 15.63 months, *P* = 0.877; OS, NR vs. NR, *P* = 0.762, for osimertinib plus RT and osimertinib groups, respectively, Fig. 3).

### Evaluation of treatment toxicities

The incidence of AEs is shown in Table 3. The rate of any grade AEs was 32.5% (13/40) in the osimertinib alone group and 47.6% (10/21) in the osimertinib plus RT group. The rate of grade 3–4 AEs was relatively higher in the osimertinib plus RT group than in the osimertinib alone group (19.0% vs. 7.5%, *P* = 0.220). However, the difference was not significant. The most common grade 3–4 AEs were decreased neutrophil count in the monotherapy group and leukoencephalopathy in the combination with radiotherapy group.

**Table 3:** Incidence of AEs

Treatment-related AEs, *n* (%)	Osimertinib group **(***n* = 40)	Osimertinib + RT group (*n* = 21)	*P*
Any grade	13 (32.5)	10 (47.6)	0.762
Fatigue	1 (2.5)	0 (0)	
Rash	5 (12.5)	1 (4.7)	
Diarrhea	3 (7.5)	1 (4.7)	
Myocardial damage	2 (5.0)	0 (0)	
Pneumonitis	3 (7.5)	0 (0)	
Oral ulcer	0 (0)	1 (4.7)	
Leukoencephalopathy	0 (0)	6 (28.5)	
Neutrophil count decreased	6 (15.0)	3 (14.2)	
Grade ≥ 3	3 (7.5)	4 (19.0)	0.220
Neutrophil count decreased	3 (7.5)	1 (4.7)	
Leukoencephalopathy	0	3 (14.2)	

*RT* cranial radiotherapy

All patients with leukoencephalopathy had received concurrent WBRT or WBRT-SIB in the osimertinib plus RT group. The incidence of leukoencephalopathy in all patients receiving WBRT or WBRT-SIB was 31.5% (6/19). According to Fazekas grade, 3 patients were grade 3, 2 patients were grade 2, and one patient was grade 1. According to the Fazekas score, 3 patients had a score equal to or higher than 5 points, 2 patients had a score of 4 points, and one patient had a score of 2 points (Table 4). In patients with leukoencephalopathy, 4 patients had EGFR 19del and 2 patients had L858R.

**Table 4:**
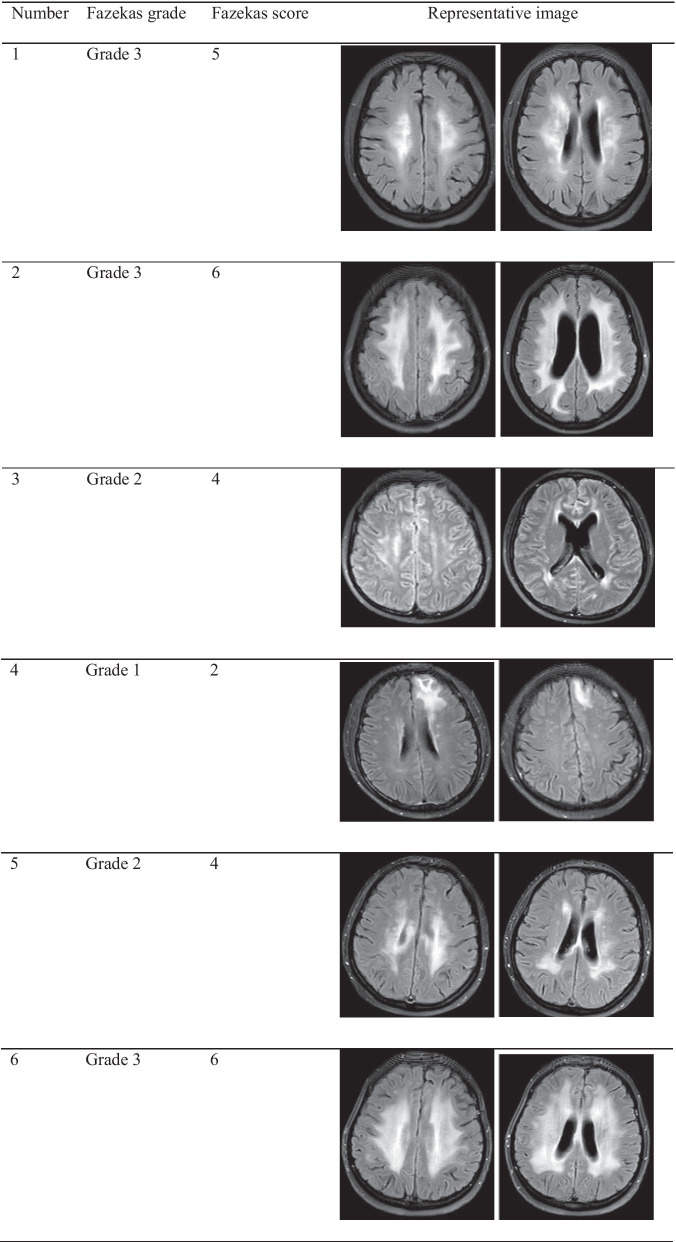
Fazekas grade and score of leukoencephalopathies

## Discussion

Osimertinib, a third-generation TKI drug, has been approved as first-line therapy for NSCLC patients harboring EGFR-positive mutations. However, it is unknown whether the CNS efficacy of osimertinib plus cranial radiotherapy is better than that of osimertinib alone in EGFR-positive NSCLC patients with brain metastases. In our study, iPFS in the osimertinib plus RT group was not superior to that in the osimertinib alone group. The intracranial DCR in the combination group was only relatively higher than that in the monotherapy group and was not statistically significant. The two groups were also not significantly different in systemic PFS or OS. However, osimertinib combined with RT might have superior OS in patients with the L858R mutation. Notably, 31.5% of patients receiving WBRT or WBRT-SIB in the osimertinib plus RT group experienced leukoencephalopathy, a late adverse event caused by brain radiotherapy, and half of them were not less than grade 3.

In contrast to 71% in AURA3 [16] and 80% in FLAURA [14], the 21.3% systemic response rate in our study was indeed much lower. There are probably three reasons for this. First, although 67.2% of patients used osimertinib as first- or second-line therapy in our study, 32.8% of patients received osimertinib as third-line or later therapy, which was different from AURA3 and FLAURA. Second, in the AURA3 study, patients with asymptomatic, stable CNS metastases who did not require glucocorticoids for at least 4 weeks were eligible [16]. Seventy-five patients with CNS lesions were enrolled in osimertinib group, and 7 patients among them had potential LM [17]. As a retrospective study, our study enrolled patients with symptomatic brain metastases and LM, accounting for 54.1% and 44.3% of all patients, respectively. These patients may have a greater tumor burden and worse general condition, achieving a lower tumor response. Moreover, in our study, 10 patients had unknown T790M status, which may also limit the therapeutic efficacy of osimertinib.

Nowadays, SRS plays an increasingly important role in radiotherapy of patients with BMs. According to the latest guideline [10], for patients with oligo-BMs, SRS was recommended as preferred radiotherapy mode. Moreover, SRS could also be considered for patients with 4–10 BMs and cumulative tumor volume less than 15 ml or after complete or incomplete resection of BMs. With the decline of therapeutic status, WBRT is mainly used for patients with multiple BMs, LM or not eligible for SRS. In patients with BMs who receive WBRT, hippocampal avoidance and memantine are recommended to better preserve cognitive function [18]. The reason for transformation in therapeutic status is mainly that SRS alone has potentially higher local control rate and less rate of neurocognitive decline on the premise of survival equal to WBRT [19, 20]. In our study, more patients received WBRT for the following four primary causes. Firstly, 61.9% of patients in the osimertinib plus RT group were more than 3 BMs. During the study enrollment period, WBRT remained to be recommended by guidelines for patients with multiple BMs. Secondly, SRS requires sophisticated equipment as the foundation. It was difficultly performed until our hospital had MR-Sim in 2020. Thirdly, 33.3% of patients had LM in the osimertinib plus RT group, and WBRT was considered for these patients. Fourthly, the relatively higher cost also limits the clinical use of SRS. Overall, as a retrospective study, the low number of SRS cases was indeed one of the major limitations of our study, and to explore the efficiency and adverse effect of SRS combination with osimertinib, large sample or prospective study is further needed.

Whether cranial radiotherapy plus TKI is more beneficial remains be controversial. A meta-analysis including 12 non-comparative observational studies suggested that upfront cranial radiotherapy could improve intracranial disease control and survival outcomes compared with TKI alone, although it was accompanied by more neurological adverse events [12]. Contrary to the above results, a retrospective analysis including 230 patients indicated that TKI plus WBRT did not have a survival benefit compared with TKI alone in NSCLC patients with BM and EGFR-positive mutations [21]. However, most previous studies comparing TKI plus RT with TKI alone in patients with NSCLC and brain metastases were based on gefitinib or erlotinib, and studies about osimertinib have rarely been reported. Our results revealed that iPFS, systemic PFS, and OS in the osimertinib plus RT group were not superior to those in the osimertinib alone group. Partially similar to our results, a retrospective study from Stanford Cancer Center suggested that receiving radiation before starting osimertinib for NSCLC patients with progressing brain metastases improved intracranial control rate but did not prolong the time to treatment failure, PFS, or OS [22]. The largest real-world study published recently exploring the clinical value of cranial radiotherapy in osimertinib-treated EGFR-mutant NSCLC with BMs showed upfront cranial RT did not significantly improve iPFS, PFS, and OS in the whole population. However, this study revealed, in patients with oligo-BMs, upfront SRS was independently associated with improved iPFS, PFS, and OS [23]. In our study, although the iPFS between our groups was not significantly different, the trends of the iPFS curve suggested that osimertinib plus RT was relatively better. The main reason for the absence of a significant difference might be the small sample size, especially in the osimertinib plus TKI group. Thus, the relevant conclusion still needs to be further verified in the future.

In the subgroup analysis, we observed that osimertinib alone resulted in longer survival than osimertinib plus RT for patients with 19 del, while in patients with L858R, osimertinib plus RT led to longer survival than osimertinib alone. One of the potential reasons was that patients with 19 del had a higher rate of the T790M mutation, achieving better efficacy when osimertinib was applied. In a study exploring the distinction of TKI resistance between patients with 19 del and L858R mutations, Wu et al. reported that the proportion of T790M mutations was 50.4% in patients with 19 del and 36.5% in patients with L858R (*P* = 0.043) [24]. Additionally, previous studies suggested that patients with 19 del were received more benefit from TKI treatment than patients with L858R [25–27]. However, the TKI drugs in their study were gefitinib, erlotinib or afatinib. Although the evidence provided by the subgroup analyses is limited, they offer a direction for future studies to select appropriate treatment modes for patients with NSCLC and BM according to distinct EGFR types.

LM, as an extremely poor prognostic factor for survival, only had a median OS of only 3–6 months in unselected NSCLC patients [28, 29]. The incidence of LM in patients harboring EGFR mutations was 7.7% higher than that in patients harboring wild-type EGFR [30]. As a prospective study relevant to osimertinib in NSCLC patients with LM, the BLOOM study indicated that osimertinib at 160 mg had an LM duration of response of 15.2 months and a median OS of 11.0 months [31, 32]. Importantly, BLOOM study found that neurologic function was improved in 57% (12/21) of patients with abnormal symptoms at baseline after receiving osimertinib at 160 mg [31]. In a series of AURA studies, 80 mg osimertinib was also beneficial for LM patients, with a median LM PFS of 11.1 months and a median OS of 18.8 months [33]. Whether patients with LM could benefit from WBRT remains controversial. In a retrospective study enrolling 109 EGFR-positive patients with LM, 42 patients with WBRT did not have longer OS than those without WBRT (9.3 months vs. 8.1 months, *P* = 0.448) [30]. Moreover, 33 patients treated with WBRT plus TKI also did not have a longer OS than those treated with TKI alone (9.7 months vs. 10.1 months, *P* = 0.778), which is consistent with our results. Currently, studies on the efficacy of osimertinib plus RT in patients with LM are lacking. Our subgroup analysis found that CNS efficacy and OS in the osimertinib plus RT group were not superior to those in the osimertinib alone group in patients with LM. As osimertinib prolongs the OS of patients with LM, the role of cranial radiotherapy may be limited. However, the timing of radiotherapy and dose of osimertinib in patients with LM still need further exploration.

Although the rate of adverse events was not significantly different between the two groups, leukoencephalopathy in the osimertinib plus RT group is worth noting. As a late adverse event after cranial radiotherapy, leukoencephalopathy could damage cerebral white matter, causing motor dysfunction, emotional change, dementia, personality change, urinary incontinence, seizure and coma. Ebi et al. observed leukoencephalopathy in 23 of 111 patients after receiving WBRT, and older age was a significant risk factor [34]. In SCLC, Mayinger et al. conducted a study to compare the risk of leukoencephalopathy after prophylactic cranial irradiation (PCI) with or without hippocampal avoidance (HA). Their result suggested that the risk of leukoencephalopathy was increased after HA-PCI compared with no HA-PCI during the follow-up time [35]. Once leukoencephalopathy has developed, there are no effective therapeutic measures. In our study, leukoencephalopathy occurred in six of nineteen patients who received WBRT, including five patients who received WBRT and one patient who received WBRT-SIB. The rate of leukoencephalopathy was up to 31.5%, which appears higher than that previously reported. A potential reason was that osimertinib may aggravate the development of leukoencephalopathy. Moreover, as osimertinib improves efficacy in patients with BM and MRI technology progresses, leukoencephalopathy may become more common and easier to discover. Given this radiation-induced adverse event in the era of osimertinib, for patients eligible for SRS, SRS should be recommended rather than WBRT.

As a retrospective analysis, there were some limitations in our study. Firstly, the small sample size could impact the statistical power and lead to no significant differences between the two groups. Due to the small sample size, the results are not representative of the whole population, and thus a study with a large sample size is needed to prove this hypothesis. Secondly, the number of patients treated with SRS is less, which may reduce the clinical reference value of our study. Thirdly, the available baseline features in our retrospective study were limited. Some important clinical information that may affect the survival time and treatment response, such as the KPS score and T790M mutation type, was not available for all patients. Fourth, as a retrospective study, because the guidelines and consensus about which patients should receive RT in patients treated with osimertinib were absent, the treatment regimens decided by different attending doctors may still have selection bias.

## Conclusions

In conclusion, our study suggested that the addition of cranial radiotherapy to osimertinib did not improve clinical outcomes compared to osimertinib alone in NSCLC patients with EGFR-positive mutations and brain metastases. However, osimertinib combined with RT may be a better choice for patients with L858R. Meanwhile, when osimertinib is combined with cranial radiotherapy, leukoencephalopathy should be noticeable. Our study provides directions for further studies and valuable clues about the treatment mode and adverse events of osimertinib with or without cranial radiotherapy in patients with EGFR-positive mutations and brain metastases.

## Data Availability

All data generated or analyzed during this study are included in this published article.

## Abbreviations

NSCLC: Non-small-cell lung cancer
BM: Brain metastases
EGFR: Epidermal growth factor receptor
BBB: Blood brain barrier
iPFS: Intracranial progression free survival
TKI: Tyrosine kinase inhibitor
RT: Cranial radiotherapy
WBRT: Whole-brain radiation therapy
SRS: Stereotactic radiosurgery
WBRT-SIB: WBRT with simultaneous integrated boost
CNS: Central nervous system
CSF: Cerebrospinal fluid
OS: Overall survival
PFS: Progression free survival
LM: Leptomeningeal metastases
19del: Exon 19 deletion
MRI: Magnetic resonance imaging
MR-Sim: Magnetic resonance simulator
CT: Contrast-enhanced computed tomography
RECIST: Response Evaluation Criteria in Solid Tumors
AEs: Adverse events
CTCAE: Common Terminology Criteria for Adverse Events
ORR: Objective response rate
CR: Complete response
PR: Partial response
DCR: Disease control rate
SD: Stable disease
